# Abcès tuberculeux de la face postérieure de la cuisse: à propos d'un cas

**DOI:** 10.11604/pamj.2014.19.50.4683

**Published:** 2014-09-22

**Authors:** Mohammed Chahbouni, Issam Eloukili, Moulay Omar Lamrani, Mohamed Kharmaz, Farid Ismail, Mustapha Mahfoud, Ahmed El Bardouni, Mohamed Saleh Berrada, Mouradh El Yaacoubi

**Affiliations:** 1 Service de Traumatologie-Orthopédie, CHU Ibn Sina, Rabat, Maroc

**Keywords:** Abcès, tuberculeuse, parties molles, anti bacillaire, abscess, tuberculosis, soft tissue, anti bacillary

## Abstract

Les auteurs rapportent le cas d'un volumineux abcès tuberculeux de la face postérieure de la cuisse droite chez une femme de 55 ans. L’étude de cette observation nous a permis d'analyser cette affection, sa fréquence, sa localisation, les moyens de son diagnostic positif et différentiel, ainsi que les mesures thérapeutiques adéquates afin de prévenir les complications qu'elle peut engendrer.

## Introduction

L'atteinte tuberculeuse est une pathologie fréquente dans les zones d'endémie tuberculeuse et n'est pas exceptionnelle dans les pays développés chez l'immigrant ou le sujet immunodéprimé.

## Patient et observation

Il s'agit d'une patiente âgée de 55 ans, opérée pour une fracture du col fémoral droit 13 ans auparavant pour laquelle elle avait bénéficié d'un vissage du col, qui a évolué vers une pseudarthrose. La patiente a refusé tout traitement. La patiente a présenté une tuméfaction indolore de la face postérieure de la cuisse droite qui a augmenté rapidement de volume. La patiente a également rapporté la notion de sueurs nocturnes, d'anorexie et de discrète altération de l’état général.

**Figure 1:**
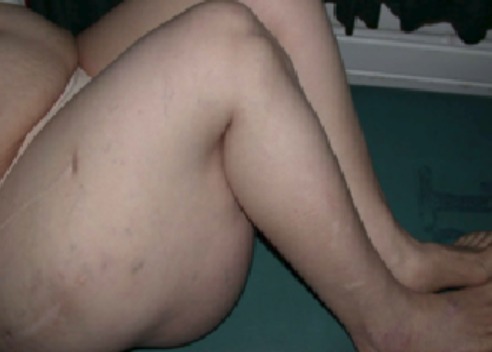
Vue de profil de la cuisse droite montrant une tuméfaction de la face postérieure

**Figure 2:**
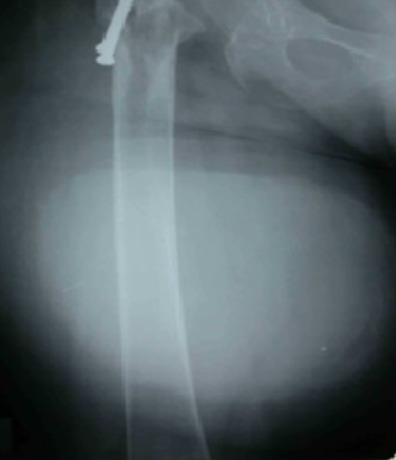
Radiographie de face de la cuisse montrant l'ombre d'une masse

**Figure 3:**
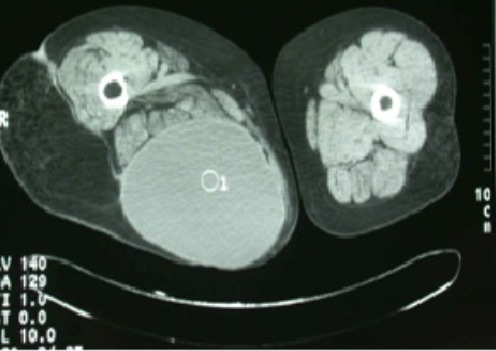
Coupe transverse du TDM montrant une collection liquidienne dans la loge postérieure de la cuisse

**Figure 4:**
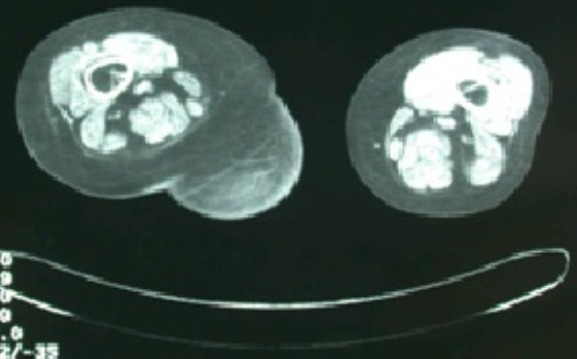
Coupe transverse du TDM montrant une collection liquidienne dans la loge postérieure de la cuisse

L'examen de la marche retrouvait une boiterie secondaire au raccourcissement du membre inférieur droit d'environ 2 centimètres. L'inspection en position debout de la patiente retrouvait une volumineuse tuméfaction de la face postérieure de la cuisse droite d'environ 30 centimètres de grand diamètre s’étendant du pli fessier inférieure jusqu'au quart inférieur de la face postérieure de la cuisse droite (Figure 1). Il s'agissait d'une tuméfaction non douloureuse, de consistance molle, sans signes inflammatoires associés et mobile par rapport au plans superficiel et profond. Le reste de l'examen était sans particularité. Par ailleurs, on a noté l'absence d'adénopathies au niveau du pli inguinal et du creux poplité. A la radiographie standard, on a retrouvé une volumineuse opacité se projetant au niveau de la face postérieure de la cuisse droite (Figure 2). L’échographie quant à elle a objectivé une image hypoechogène homogène. Le bilan a été complété par une TDM qui a montré une masse hypodense de tonalité liquidienne affleurant le plan sous cutané (Figure 3, Figure 4).

L'abord chirurgical de la masse a découvert un abcès superficiel, de contenu jaunâtre. L'intervention a consisté en l'ablation de la gaine de l'abcès qui se logeait entre le plan sous cutané et l'aponévrose de la loge postérieure de la cuisse droite et qui s’étendait à travers les interstices musculaires jusqu'au sacrum. L’étude anatomopathologique a confirmé la présence de granulomes épithelio-giganto-cellulaires avec nécrose caséeuse. Ce traitement chirurgical a été complété par un traitement médical à base d'anti-bacillaires durant 9 mois. Les suites postopératoires ont été simples et l’évolution a été favorable.

## Discussion

L'abcès tuberculeux de la cuisse est très rare [1]. Le plus souvent, il est étudié dans le cadre des manifestations cliniques de la tuberculose ostéo-articulaire de la hanche [1–3]. Il survient vers les quatrièmes et les cinquièmes décades [2–4]. Il s'observe plus chez l'homme. Dans les antécédents, la notion de traumatisme est largement soulignée dans toutes les études [3–5]. Il semble que le traumatisme crée des altérations tissulaires capables, soit de fixer localement un bacille tuberculeux, soit de réveiller un foyer quiescent. Dans la majorité des études réalisées, on note la notion d'antécédents tuberculeux [4–6].

Le début est souvent insidieux entraînant un retard de diagnostic. La phase d’état est marquée par une tuméfaction des parties molles réalisant un abcès froid de volume variable [1, 2]. Une douleur de la hanche et des limitations de mouvement peuvent s'associer [2–4], la fistulisation est la conséquence d'une évolution tardive. L’état général peut être altéré ou conservé et l'existence d'une amyotrophie de la cuisse est variable.

Sur le plan biologique la vitesse de sédimentation et la numération formule sanguine sont habituellement normales. L'intradermo-réaction à la tuberculine est positive. Après ponction à l'aiguille d'un abcès collecté et coloration de ZEIHL, l'examen direct est positif mais ne permet pas de différencier entre les myobactéries tuberculeux et atypiques [5–7]. Cette précision sera apportée par la culture en milieu de LOWENSTEIN et JENSEN où le bacille pousse lentement en 21 à 72 jours, d'où la nécessité de répéter les cultures. L'examen histologique sur les différents prélèvements permet de mettre en évidence le plus souvent des follicules épithelio-giganto-cellulaire avec nécrose caséeuse [5–7]. La radiographie standard du bassin de face et du fémur peuvent être sans particularités, mais le plus souvent on retrouve une opacité des parties molles de volume variable avec atteinte osseuse inconstante. L’échographie montre des collections liquidiennes à contenu hétérogène. La TDM définit le degré d'extension. La fistulographie est employé en cas de fistule distale. La scintigraphie cherche une atteinte osseuse débutante [7–9].

Les principaux diagnostics différentiels sont l'abcès à pyogènes, à myobactéries atypique, le kyste hydatique, et certaines tumeurs. Le traitement chirurgical est basé sur le drainage et la résection de la poche de l'abcès avec un lavage abondant ou des ponctions à répétitions. Le traitement est médical à base des antibacillaires [10–12] par l'association de 3 médicaments à la phase initiale qui dure 2 mois (Rifampicine, Pyrazinamide, et Streptomycine) et 2 médicaments à la phase d'entretien (Rifampicine, Pyrazinamide) qui dure 4 mois. L’évolution est attestée par des critères cliniques, biologiques, et radiologiques.

## Conclusion

Tout abcès des parties molles doit évoquer l'origine tuberculeuse. La recherche étiologique doit réunir un fuseau d'arguments anamnestiques, cliniques, et paracliniques. Le traitement quant à lui doit être adéquat afin d’éviter l’évolution vers les complications.
