# Corrigendum: Abcès tuberculeux de la face postérieure de la cuisse: à propos d’un cas

**DOI:** 10.11604/pamj.2018.30.104.15871

**Published:** 2018-06-06

**Authors:** Mohammed Chahbouni, Issam Eloukili, Moulay Omar Lamrani, Mohamed Kharmaz, Farid Ismail, Mustapha Mahfoud, Ahmed El Bardouni, Mohamed Saleh Berrada, Mouradh El Yaacoubi

**Affiliations:** 1 Service de Traumatologie-Orthopédie, CHU Ibn Sina, Faculté de Médecine et de Pharmacie de Rabat, Université Mohamed V, Rabat, Maroc

**Keywords:** Corrigendum, Abcès, tuberculeuse, parties molles, anti bacillaire

## Abstract

This corrigendum corrects article “Abcès tuberculeux de la face postérieure de la cuisse: à propos d’un cas. The Pan African Medical Journal. 2014;19:50. doi:10.11604/pamj.2014.19.50.4683.

## Corrigendum

The author affiliation in the published article [1] “Service de Traumatologie-Orthopédie, CHU Ibn Sina, Rabat, Maroc” is incomplete. It should be read: “Service de Traumatologie-Orthopédie, CHU Ibn Sina, Faculté de Médecine et de Pharmacie de Rabat, Université Mohamed V, Rabat, Maroc”.
